# Novel (*E*)-β-Farnesene Analogues Containing 2-Nitroiminohexahydro-1,3,5-triazine: Synthesis and Biological Activity Evaluation

**DOI:** 10.3390/molecules21070825

**Published:** 2016-06-24

**Authors:** Yaoguo Qin, Jingpeng Zhang, Dunlun Song, Hongxia Duan, Wenhao Li, Xinling Yang

**Affiliations:** 1 Department of Applied Chemistry, College of Science, China Agricultural University, Beijing 100193, China; qyg@cau.edu.cn (Y.Q.); jpzhang@cau.edu.cn (J.Z.); hxduan@cau.edu.cn (H.D.); oliverlwh@cau.edu.cn (W.L.); 2 Department of Entomology, College of Plant Protection, China Agricultural University, Beijing 100193, China; songdl@cau.edu.cn

**Keywords:** (*E*)-β-farnesene analogues, 2-nitroiminohexahydro-1,3,5-triazine, synthesis, bioactivity, structure-activity relationship

## Abstract

In order to discover novel eco-friendly compounds with good activity for aphid control, (*E*)-β-farnesene (EβF), the main component of the aphid alarm pheromone, was chosen as the lead compound. By introducing a 2-nitroimino-hexahydro-1,3,5-triazine moiety (abbreviated NHT) to replace the unstable conjugated double bond system of EβF, a series of novel EβF analogues containing the NHT moiety were synthesized via the reaction of substituted NHT rings with (*E*)-1-chloro-3,7-dimethylocta-2,6-diene. All the compounds were characterized by ^1^H-NMR, ^13^C-NMR, IR, and high resolution mass spectroscopy (HRMS). The bioassay results showed that all the analogues displayed different repellent and aphicidal activities against green peach aphid (*Myzus persicae*). Particularly, the analogue **4r** exhibited obvious repellent activity (repellent proportion: 78.43%) and similar aphicidal activity against *M. persicae* (mortality: 82.05%) as the commercial compound pymetrozine (80.07%). A preliminary structure-activity relationship (SAR) study was also performed, which offered valuable clues for the design of further new EβF analogues.

## 1. Introduction

As some of the major pests in agriculture, aphids cause considerable damage [1,2] to crop growth, yield and quality by sucking phloem sap [3], secreting honeydew to induce frequently plant sooty moulds [1,2,4] and transmitting plant viruses [5,6,7]. Owing to the variety of species, large population, fast reproduction and high resistance to chemical insecticides, aphid control has become more and more challenging. As the idea of eco-friendly pest control is increasingly recognized worldwide, the strategy for aphid control has changed from traditional “killing” to rational “regulation” [8]. In the past decades, traditional aphid control methods have largely relied on agrochemicals, which has led to increasing pest resistance and cross resistance. Therefore, it is important to develop novel aphid control strategies. Using aphid alarm pheromones could be an alternative way to control their population by manipulating aphid behaviors, and is also regarded to be conducive to ecological protection [9,10].

**Scheme 1:**
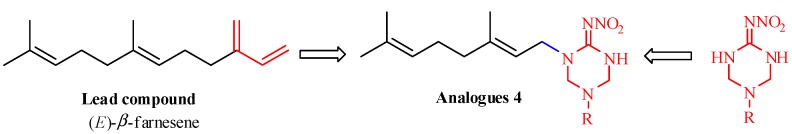
The design strategy of EβF analogues **4**.

Aphid alarm pheromone is a strong and efficient pheromone produced and utilized by most aphid species [11,12,13,14]. The main component of aphid alarm pheromone is (*E*)-β-farnesene (EβF, (*E*)-7,11-dimethyl-3-methylenedodeca-1,6,10-triene, Scheme 1), which possesses multiple biological functions. Besides its alarm activity, EβF also showed insecticidal activity at high doses [15], and could be used as the synergistic agent when mixed with commercial insecticides to control aphids [16]. However, it is difficult to use in the field because of its instability due to the presence of a conjugated double bond system. Therefore, it is necessary to develop novel EβF analogues with good stability for aphid management.

With the advantages of multiple bioactivities, heterocycles, such as triazine, pyrazole, pyridine, oxadiazine and triazole, play important roles in the development of the agrochemical and medical fields [17]. Among them, the 2-nitroiminohexahydro-1,3,5-triazine (NHT) system, with its simple structure and low cost, is a very important active group in the agrochemically active molecule field. Compounds containing NHT have been discovered to possess a wide range of insecticidal properties, such as inhibiting population growth of *Aphis gossypii* [18], *Myzus persicae* [19], *Nephotettix cincticeps* [20], *Ctenocephalides felies* [21], *Aphis medicagini* [22], *Spodoptera littoralis* [23], and *Nilaparvata lugens* [24].

Introducing active groups is an effective method to develop new eco-friendly agrochemicals. As mentioned above, 2-nitroiminohexahydro-1,3,5-triazine is a very important and active group widely existing in agrochemicals, especially in insecticides. In order to develop alarm pheromone analogues with good activity and high stability for eco-friendly aphid management, herein a series of EβF analogues were designed and synthesized by newly introducing an effective group, NHT, to replace the conjugated double bonds of EβF. The design strategy of these EβF analogues is shown in Scheme 1. Their biological activities, including repellent activity as well as aphicidal activity, were investigated. Their structure-activity relationships were also studied to provide some useful clues for the further design and development of novel EβF analogues.

## 2. Results and Discussion

### 2.1. Chemistry

**Scheme 2:**
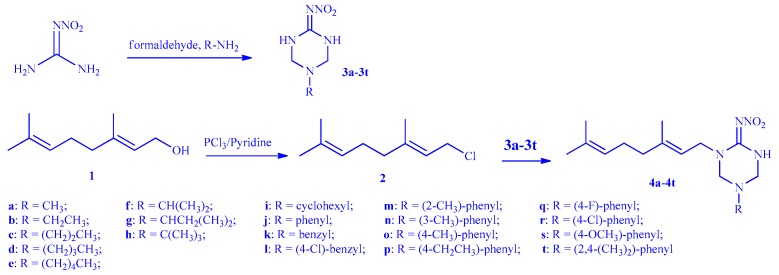
Synthetic route to the EβF analogues **4a**–**4t**.

The synthetic route for the intermediates and EβF analogues is illustrated in Scheme 2. The intermediate **2** (geranyl chloride) was obtained from geraniol (**1**) and phosphorus trichloride (PCl_3_) via a nucleophilic substitution reaction using pyridine as an acid-binding agent. According to the reported method [25], the key 5-substituted-1,3,5-triazine-2-*N*-nitrate amine intermediates **3** were prepared by Mannich reactions between nitroguanidine, formaldehyde and different commercially available primary amines in a one-pot method using a protic solvent. Under moderate conditions, the EβF analogues **4** were synthesized through the nucleophilic substitution reaction between intermediates **2** and **3a**–**3t** in acetonitrile using K_2_CO_3_ as acid acceptor to produce the corresponding EβF analogues **4a**–**4t**. The synthetic procedure is described in Section 3.2.3.

The structures of all synthesized EβF analogues **4a**–**4t** were confirmed by IR, ^1^H-NMR, ^13^C-NMR and HR-ESI-MS. Their physical and chemical properties and structure characterization were described in Section 3.2.3. In the IR spectra, the analogues showed strong absorptions around 3300 cm^−1^ due to the N-H stretching vibration. Strong bands at about 1600 cm^−1^ were detected because of the C=C stretching vibration. The extremely strong absorption bands detected around 1550 and 1370 cm^−1^ are due to the nitro groups.

In the ^1^H-NMR spectrum of EβF analogues **4a**–**4t**, a wide single peak in the δ 9.48–9.75 ppm chemical shift range was due to the presence of NH protons. The protons of two double bonds were observed at δ 5.10–5.22 ppm in compounds **4a**–**4i** and **4k**–**4l** while the signals shifted upfield to about δ 4.94–5.07 ppm in analogues **4j** and **4m**–**4t**. The signals of the C-H protons in NHT were clearly observed at δ 4.33–4.97 ppm. The protons of the methylene connected to the triazine group were split into a doublet in the range from 3.94 to 4.12 ppm with a coupling constant of 6.83–7.27 Hz attributed to the long range coupling with the proton on the adjacent carbon atom.

**Figure 1:**
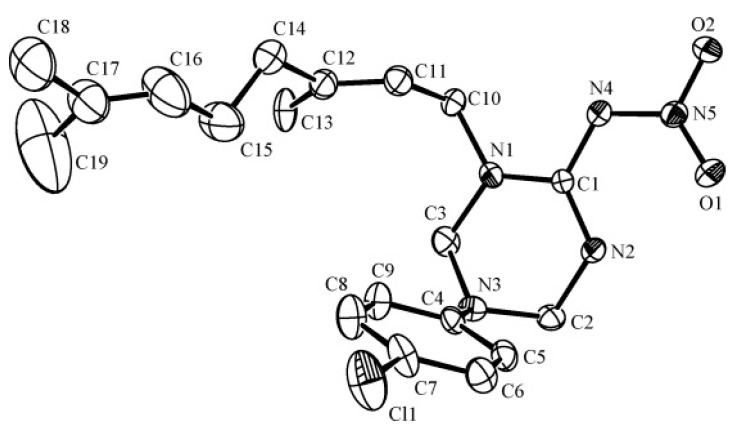
The crystal structure of the analogue **4r**.

The structure of the analogue **4r** was further confirmed by single crystal X-ray analysis. Its molecule crystal structure had been deposited with the Cambridge Crystallographic Data Centre, under deposition number CCDC 1437627. A perspective view of the compound is shown in Figure 1 and the crystal data are presented in Table 1. Crystal Data for **4r** (C_19_H_26_ClN_5_O_2_, M = 391.90): monoclinic, space group P21/c (No. 14), a = 5.8324(11) Å, b = 38.200(5) Å, c = 9.1298(13) Å, β = 94.170(15)°, V = 2028.7(5) Å^3^, Z = 4, T = 180.01(10) K, μ (Mo Kα) = 0.212 mm^−1^, Dcalc = 1.283 g/mm^3^, 8970 reflections measured (6.182 ≤ 2Θ ≤ 52.044), 3971 unique (Rint = 0.1078) which were used in all calculations. The final R1 was 0.0893 (I > 2σ (I)) and wR2 was 0.2328. More data of this crystal structure, such as the crystal data and structure refinement, crystal packing, fractional atomic coordinates (×10^4^) and equivalent isotropic displacement parameters (Å^2^ × 10^3^), bond length and bond angle of the analogue **4r** are presented in the Supplementary Materials.

In particular, the stability of **4r** and EβF was detected by high performance liquid chromatography (HPLC) after leaving them at room temperature and exposing them to air for periods up to 48 h. On such conditions, no degradation of the analogue **4r** could be detected. The EβF degraded from 92.5% to an undetected level, which was in good agreement with the reported degradation rate (94%) of EβF [26]. These results suggested that **4r** was more stable than EβF.

### 2.2. Biological Activity

#### 2.2.1. Repellent Activity

**Figure 2:**
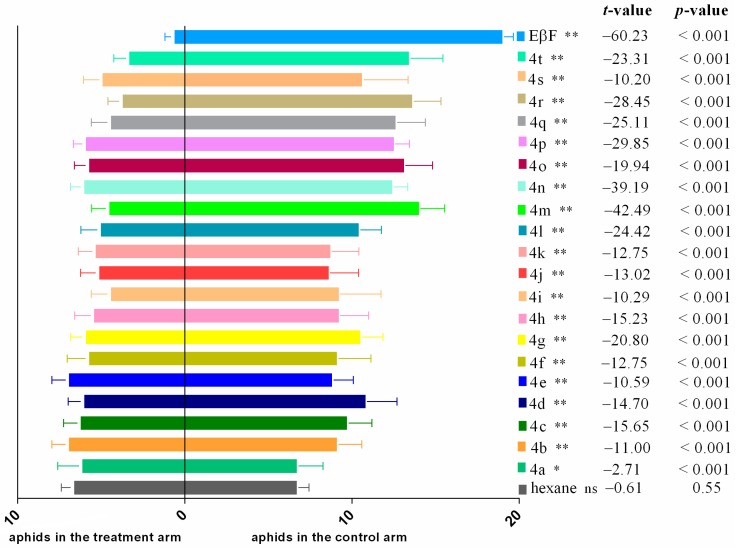
Behavioral response of *M. persicae* to EβF analogues **4a**–**4t**. The number of aphids (mean ± standard deviation) in both arms were counted and analyzed by paired *t*-test. “ns” represents no significance; “*” represents significant difference at *p* ≤ 0.05 level; and “**” represents significant difference at *p* ≤ 0.01 level.

As one of the major aphid pests in agriculture, *Myzus persicae* was chosen to test the repellent activity with a two-way olfactometer as described in Section 3.5.1. In order to determine if an EβF analogue had repellent effect, the numbers of aphids in the control and treatment arms was compared statistically with SPSS Statistics version 21 (IBM, Armonk, NY, USA) by paired t-test. Firstly, the behavioral response of apterous adult *Myzus persicae* to the solvent *n*-hexane was tested, in which aphids always showed no preference for either arm. Then, the repellent effect of these EβF analogues was measured using EβF as positive control. All there EβF analogues exhibited significant repellent differences against *M. persicae* between the mean number of aphids in control arm and those in treatment arm (Figure 2). The different EβF analogues differed in their repellent activities (Table 2). Even though some compounds displayed a fairly high repellent activity (up to 80.07% of aphids were repelled), all tested EβF analogues repelled fewer aphids than EβF itself. Generally, the EβF analogues where R was a substituted aromatic ring had a higher repellent activity than those with alkyl R groups. When R was alkyl, chain length (number of C < 4) had a positive effect on repellent activity (**4a** < **4b** < **4c** < **4d**). However, the number of chain branches seemed to have no effect on repellent activity (**4c** ≈ **4f**, **4d** ≈ **4g** ≈ **4h**). In addition, these analogues exhibited similar activity whether the R group was a benzyl ring or a phenyl ring (**4j** ≈ **4k**). A preliminary structure-activity relationship analysis discovered that the analogues with R was a substituted phenyl ring presented higher activity than when R was an unsubstituted phenyl ring. Substituent in the *ortho*-position of the phenyl were more beneficial for activity compared with *meta*- and *para*-substituents (**4i** > **4j**, **4i** > **4k**). In the same substituent position on the phenyl ring, the electron-withdrawing substituents contributed to better repellent activity than that of electron-donating groups (**4q**, **4r** > **4o**, **4p** and **4s**).

#### 2.2.2. Aphicidal Activity

Previous publications have indicated that EβF showed aphicidal activity at high doses [15], and many compounds with the NHT moiety exhibit high insecticidal activities against major pests [18,27], such as aphids, plant hoppers, spider mites and other piercing-sucking mouthparts insects. To further study the bioactivity of our synthesized EβF analogues, the aphicidal activity was preliminarily evaluated in vivo at the concentration 300 μg/mL according to the reported procedure [28].

The results are shown in Table 3. After replacing the EβF conjugated double bonds with NHT, all the analogues displayed aphicidal activity against *Myzus persicae*, and most analogues had better activity than the lead compound EβF. Particularly, the analogues **4c**–**4e**, and **4p**–**4s**, with mortalities of more than 80%, exhibited similar activity as the commercial insecticide pymetrozine (81%). Furthermore, a significant difference in mortality was observed between the analogue **4c** and pymetrozine, like between the analogue **4q** and pymetrozine. Preliminary structure-activity relationship analysis found that when R was a straight alkyl chain (number of C < 3), chain length had a positive effect on aphicidal activity (**4a** < **4b** < **4c**). However, the number of chain branches seems to give negative effect on aphicidal activity. For instance, there were a significant difference (*p* < 0.05) in the mortality between **4c** and **4f**, **4d** and **4g**, and **4d** and **4h**. The analogues where R was a cyclohexyl, unsubstituted phenyl or benzyl group exhibited lower aphicidal activity than those where R was a straight chain. Substituents in the phenyl *para*-position were beneficial for activity compared with *meta*- and *ortho*-substituents (**4o** > **4m** ≈ **4n**) and the analogues where R was either monosubstituted or bisubstituted on the phenyl ring showed good inhibition against aphids.

## 3. Materials and Methods

### 3.1. General Information

Melting points of the EβF analogues were determined on a Cole-Parmer apparatus equipped with an uncorrected thermometer (Shanghai precision instrument and Meter Co., Ltd., Shanghai, China). IR spectra were recorded on neat samples on an IR-435 spectrophotometer (Shimadzu, Kyoto, Japan) using KBr pellets. ^1^H-NMR spectra (300 MHz) and ^13^C-NMR spectra (75 MHz) were recorded on an Avance DPX300 spectrometer (Bruker, Karlsruhe, Germany). ^1^H-NMR chemical shifts are reported in δ (ppm) relative to the signal of tetramethylsilane (TMS) as internal standard, using CDCl_3_ as solvent. The ^13^C-NMR chemical shifts (δ) were reported in parts per million using the solvent peak as reference. High resolution mass spectra were determined under electron impact (150 eV) conditions using a Bruker APEX IV instrument. Silica gel (Merck 60, 230–300 mesh, Qingdao Haiyang Chemical Co., Ltd., Qingdao, China) was used for column chromatography with petroleum ether and ethyl acetate as eluents. All starting materials, reagents and solvents were analytical grade reagents and were commercially available, and all solvents were purified and dried before used.

### 3.2. Synthesis of EβF Analogues ***4a***–***4t***

#### 3.2.1. General Procedure for the Preparation of Intermediate **2**

Intermediate **2** was synthesized using the published method [8]. Briefly, phosphorus trichloride (650 mmol) was added to dried anhydrous pyridine (325 mmol) and dried *n*-hexane (100 mL) in a 500 mL round bottom flask. After the mixture was cooled to 0 °C in ice-salt bath, geraniol (16.23 mmol) and dried *n*-hexane (20 mL) was dropwise added to the mixture between 0 °C and 5 °C. The mixture was stirred for 30 min under −5 °C followed by washing with NaCl saturated solution (1 × 30 mL) and *n*-hexane (3 × 30 mL). The organic phase was combined and separately washed with saturated NaCl solution and saturated NaHCO_3_ solution until the pH = 7.0. After dried with anhydrous sodium sulfate, the organic phase was concentrated under reduced pressure to give the intermediate **2** (yellow liquid, yield 87.5%).

#### 3.2.2. General Procedure for the Preparation of Intermediate **3a**–**3t**

On the basis of reported method [25], the intermediates **3** containing different R substituents at the 5-position of NHT were prepared by the following general procedure: in a 100 mL round bottom flask, each different commercial primary amine (58 mmol), nitroguanidine (48 mmol) and 37% formaldehyde (120 mmol) were dissolved in ethanol (20 mL). The reaction mixture was stirred at 60 °C for 3 h and then was cooled to room temperature. After filtering the solid product was washed with water and acetone, respectively, and dried to give the white solid intermediates **3a**–**3t**, which were used without further purification for the synthesis of the EβF analogues **4a**–**4t**.

#### 3.2.3. General Procedure of EβF Analogues **4a**–**4t**

All the EβF analogues were synthesized according to the reported method [29]. Intermediate **2** (2.58 mmol), intermediate **3** (2.58 mmol) as prepared in Section 3.2.1 and Section 3.2.2 and dried K_2_CO_3_ (2.58 mmol) were added to dried acetonitrile (20 mL) in a 100 mL round bottom flask. The mixture was refluxed for 5 h and then cooled to room temperature, filtered and concentrated under reduced pressure. The residue was purified via silica gel column chromatography (petroleum ether/ethyl acetate = 4:1) to give the EβF analogues **4a**–**4t**. The yields, physicochemical properties and structural characterization data of **4a**–**4t** were as follows:

*N-(1-((E)-3,7-Dimethylocta-2,6-dien-1-yl)-5-methyl-1,3,5-triazinan-2-ylidene)nitramide* (**4a**), yellow wax, m.p. 45–47 °C, yield 30.5%. IR(KBr), ν/cm^−1^: 3292, 2924, 1589, 1541, 1448, 1381, 1313, 1121; ^1^H-NMR (300 MHz, CDCl_3_): δ ppm 9.57 (brs, 1H, Het-NH), 5.13–5.18 (m, 1H, C=CH), 5.02–5.06 (m, 1H, C=CH), 4.33 (s, 2H, Het-H), 4.19 (d, 2H, *J* = 4.41 Hz, Het-H), 4.07 (d, 2H, *J* = 7.13 Hz, Het-CH_2_), 2.56 (s, 3H, N-CH_3_), 2.01–2.12 (m, 4H, C-CH_2_CH_2_-C), 1.64–1.69 (m, 6H, C-(CH_3_)_2_), 1.60 (s, 3H, C-CH_3_); ^13^C-NMR (75 MHz, CDCl_3_): 154.42, 141.00, 131.37, 123.25, 117.55, 66.55, 61.71, 43.51, 39.10, 38.97, 25.83, 25.23, 17.25, 15.83. HRMS (ESI^+^) *m*/*z* calcd for C_14_H_26_N_5_O_2_, 296.20810 [M + H]^+^; found: 296.20782.

*N-(1-((E)-3,7-Dimethylocta-2,6-dien-1-yl)-5-ethyl-1,3,5-triazinan-2-ylidene)nitramide* (**4b**), white wax, m.p. 40C–42 °C, yield 39.2%. IR(KBr), ν/cm^−1^: 3275, 2969, 2925, 2856, 1597, 1556, 1380, 1239; ^1^H-NMR (300 MHz, CDCl_3_): δ ppm 9.54 (brs, 1H, Het-NH), 5.15–5.16 (m, 1H, C=CH), 5.05–5.06 (m, 1H, C=CH), 4.37–4.38 (m, 2H, Het-H), 4.24 (s, 2H, Het-H), 4.05 (d, 2H, *J* = 7.08 Hz, Het-CH_2_), 2.73 (q, 2H, *J* = 7.18 Hz, N-CH_2_), 2.05–2.10 (m, 4H, C-CH_2_CH_2_-C), 1.68–1.70 (m, 6H, C-(CH_3_)_2_), 1.60 (s, 3H, C-CH_3_), 1.14 (t, 3H, *J* = 7.22 Hz, C-CH_3_); ^13^C-NMR (75 MHz, CDCl_3_): 154.74, 141.08, 131.48, 123.26, 117.60, 64.31, 59.61, 44.64, 43.44, 39.16, 25.91, 25.29, 17.28, 15.90, 12.67. HRMS (ESI^+^) *m/z* calcd for C_15_H_28_N_5_O_2_, 310.22375 [M + H]^+^; found: 310.22324.

*N-(1-((E)-3,7-Dimethylocta-2,6-dien-1-yl)-5-propyl-1,3,5-triazinan-2-ylidene)nitramide* (**4c**), white solid, m.p. 72–73 °C, yield 38.2%. IR(KBr), ν/cm^−1^: 3274, 2959, 2932, 2874, 1587, 1556, 1375, 1288; ^1^H-NMR (300 MHz, CDCl_3_): δ ppm 9.55 (brs, 1H, Het-NH), 5.12–5.17 (m, 1H, C=CH), 5.05–5.06 (m, 1H, C=CH), 4.35–4.36 (m, 2H, Het-H), 4.22 (s, 2H, Het-H), 4.05 (d, 2H, *J* = 7.14 Hz, Het-CH_2_), 2.62 (t, 2H, *J* = 7.44 Hz, N-CH_2_), 2.05-2.10 (m, 4H, C-CH_2_CH_2_-C), 1.68–1.70 (m, 6H, C-(CH_3_)_2_), 1.60 (s, 3H, CH_3_), 0.94 (t, 3H, *J* = 7.38 Hz, C-CH_3_); ^13^C-NMR (75 MHz, CDCl_3_): 154.75, 141.03, 131.44, 123.25, 117.60, 64.80, 60.14, 52.36, 43.44, 39.16, 25.95, 25.27, 20.68, 17.27, 15.90, 11.07. HRMS (ESI^+^) *m/z* calcd for C_16_H_30_N_5_O_2_, 324.23940 [M + H]^+^; found: 324.23911.

*N-(1-((E)-3,7-Dimethylocta-2,6-dien-1-yl)-5-butyl-1,3,5-triazinan-2-ylidene)nitramide* (**4d**), white solid, m.p. 44–46 °C, yield 49.2%. IR ν/cm^−1^: 3276, 2963, 2930, 2861, 1587, 1556, 1536, 1392, 1290, 1237; ^1^H-NMR: δ ppm 9.52 (brs, 1H, Het-NH), 5.13–5.18 (m, 1H, C=CH), 5.04–5.08 (m, 1H, C=CH), 4.38 (s, 2H, Het-H), 4.23 (s, 2H, Het-H), 4.05 (d, 2H, *J* = 7.11 Hz, Het-CH_2_), 2.66 (t, 2H, *J* = 6.90 Hz, N-CH_2_), 2.06–2.10 (m, 4H, C-CH_2_CH_2_-C), 1.70 (s, 3H, C-CH_3_), 1.68 (s, 3H, C-CH_3_), 1.60 (s, 3H, C-CH_3_), 1.43–1.52 (m, 2H, C-CH_2_-C), 1.30–1.40 (m, 2H, C-CH_2_-C), 0.93 (t, 3H, *J* = 7.20 Hz, C-CH_3_); ^13^C-NMR: δ ppm 154.81, 141.16, 131.55, 123.24, 117.57, 64.80, 60.18, 50.30, 43.53, 39.21, 29.58, 26.00, 25.31, 19.77, 17.30, 15.97, 13.48. HRMS (ESI^+^) *m/z* calcd for C_17_H_32_N_5_O_2_, 338.25505 [M + H]^+^; found: 338.25464.

*N-(1-((E)-3,7-Dimethylocta-2,6-dien-1-yl)-5-pentyl-1,3,5-triazinan-2-ylidene)nitramide* (**4e**), white solid, m.p. 49–50 °C, yield 30.1%. IR ν/cm^−1^: 3293, 2955, 2929, 2858, 1587, 1541, 1428, 1395, 1303, 1238; ^1^H-NMR: δ ppm 9.52 (brs, 1H, Het-NH), 5.12–5.17 (m, 1H, C=CH), 5.03–5.07 (m, 1H, C=CH), 4.38 (s, 2H, Het-H), 4.22 (s, 2H, Het-H), 4.05 (d, 2H, *J* = 7.14 Hz, Het-CH_2_), 2.64 (t, 2H, *J* = 7.14 Hz, N-CH_2_), 2.04–2.09 (m, 4H, C-CH_2_CH_2_-C), 1.69 (s, 3H, C-CH_3_), 1.68 (s, 3H, C-CH_3_), 1.60 (s, 3H, C-CH_3_), 1.44–1.54 (m, 2H, C-CH_2_-C), 1.28–1.34 (m, 4H, C-CH_2_CH_2_-C), 0.90 (t, 3H, *J* = 6.93 Hz, C-CH_3_); ^13^C-NMR: δ ppm 154.78, 141.15, 131.55, 123.24, 117.56, 64.79, 60.16, 50.57, 43.51, 39.21, 28.78, 27.19, 25.99, 25.32, 22.10, 17.32, 15.98, 13.62. HRMS (ESI^+^) *m/z* calcd for C_18_H_34_N_5_O_2_, 352.27070 [M + H]^+^; found: 352.27066.

*N-(1-((E)-3,7-Dimethylocta-2,6-dien-1-yl)-5-isopropyl-1,3,5-triazinan-2-ylidene)nitramide* (**4f**), white solid, m.p. 64–66 °C, yield 22.5%. IR ν/cm^−1^: 3294, 2968, 2923, 2854, 1587, 1552, 1384, 1290; ^1^H-NMR: δ ppm 9.51 (brs, 1H, Het-NH), 5.18–5.19 (m, 1H, C=CH), 5.05–5.06 (m, 1H, C=CH), 4.45–4.46 (m, 2H, Het-H), 4.32 (s, 2H, Het-H), 4.05 (d, 2H, *J* = 7.17 Hz, Het-CH_2_), 2.88–3.07 (m, 1H, C-CH-C), 2.06–2.10 (m, 4H, C-CH_2_CH_2_-C), 1.68–1.71 (m, 6H, C-(CH_3_)_2_), 1.58–1.60 (m, 3H, C-CH_3_), 1.16 (s, 3H, C-CH_3_) , 1.14 (s, 3H, C-CH_3_); ^13^C-NMR: δ ppm 155.39, 141.04, 131.47, 123.26, 117.62, 62.62, 57.57, 48.21, 47.34, 43.44, 39.18, 25.94, 25.29, 20.80, 18.82, 17.28, 15.91. HRMS (ESI^+^) *m/z* calcd for C_16_H_30_N_5_O_2_, 324.23940 [M + H]^+^; found: 324.23914.

*N-(1-((E)-3,7-Dimethylocta-2,6-dien-1-yl)-5-isobutyl-1,3,5-triazinan-2-ylidene)nitramide* (**4g**), white solid, m.p. 87–88 °C, yield 52.8%. IR ν/cm^−1^: 3279, 2961, 2926, 2871, 2853, 1587, 1554, 1535, 1380, 1289, 1239; ^1^H-NMR: δ ppm 9.56 (brs, 1H, Het-NH), 5.15–5.20 (m, 1H, C=CH), 5.06–5.10 (m, 1H, C=CH), 4.36 (s, 2H, Het-H), 4.22 (s, 2H, Het-H), 4.07 (d, 2H, *J* = 7.11 Hz, Het-CH_2_), 2.45 (d, 2H, *J* = 7.26 Hz, N-CH_2_), 2.08–2.12 (m, 4H, C-CH_2_CH_2_-C), 1.74 (s, 3H, C-CH_3_), 1.71 (s, 3H, C-CH_3_), 1.70 (s, 1H, C-CH), 1.62 (s, 3H, C-CH_3_), 0.96 (s, 3H, C-CH_3_), 0.94 (s, 3H, C-CH_3_); ^13^C-NMR: δ ppm 154.91, 141.22, 131.60, 123.24, 117.53, 65.44, 60.83, 58.87, 43.57, 39.23, 26.56, 26.04, 25.34, 20.05, 17.32, 16.00. HRMS (ESI^+^) *m/z* calcd for C_17_H_32_N_5_O_2_, 338.25505 [M + H]^+^; found: 338.25476.

*N-(1-((E)-3,7-Dimethylocta-2,6-dien-1-yl)-5-(tert-butyl)-1,3,5-triazinan-2-ylidene)nitramide* (**4h**), white solid, m.p. 93–95 °C, yield 40.3%. IR ν/cm^−1^: 3297, 2965, 2923, 2877, 1595, 1553, 1365, 1332; ^1^H-NMR: δ ppm 9.56 (brs, 1H, Het-NH), 5.22–5.26 (m, 1H, C=CH), 5.05–5.07 (m, 1H, C=CH), 4.52 (brs, 2H, Het-H), 4.36 (brs, 2H, Het-H), 4.08 (d, 2H, *J* = 7.26 Hz, C=CH), 2.03–2.09 (m, 4H, C-CH_2_CH_2_-C), 1.68–1.72 (m, 6H, C-(CH_3_)_2_), 1.46–1.48 (m, 3H, CH_3_), 1.21 (s, 9H, C(CH_3_)_3_); ^13^C-NMR: δ ppm 155.61, 140.99, 131.46, 123.27, 117.66, 60.56, 55.63, 54.37, 43.28, 39.17, 27.98, 25.94, 25.29, 17.28, 15.89. HRMS (ESI^+^) *m/z* calcd for C_17_H_32_N_5_O_2_, 338.25505 [M + H]^+^; found: 338.25485.

*N-(1-((E)-3,7-Dimethylocta-2,6-dien-1-yl)-5-cyclohexyl-1,3,5-triazinan-2-ylidene)nitramide* (**4i**), white solid, m.p. 99–100 °C, yield 63.8%. IR ν/cm^−1^: 3287, 2926, 2853, 1586, 1554, 1536, 1383, 1240; ^1^H-NMR: δ ppm 9.50 (brs, 1H, Het-NH), 5.15–5.20 (m, 1H, C=CH), 5.04–5.06 (m, 1H, C=CH), 4.46–4.47 (m, 2H, Het-H), 4.33 (s, 2H, Het-H), 4.05 (d, 2H, *J* = 7.17 Hz, Het-CH_2_), 2.64–2.65 (m, 1H, 1-cyclohexyl-H), 2.01–2.10 (m, 4H, C-CH_2_CH_2_-C), 1.77–1.91 (m, 4H, 2,6-cyclohexyl-H), 1.68–1.71 (m, 6H, C-(CH_3_)_2_), 1.60 (s, 3H, CH_3_), 1.14–1.30 (m, 6H, 3,4,5-cyclohexyl-H); ^13^C-NMR: δ ppm 155.36, 141.03, 131.46, 123.24, 117.60, 62.21, 57.16, 56.02, 43.37, 39.19, 30.71, 25.98, 25.29, 25.20, 24.56, 17.28, 15.94. HRMS (ESI^+^) *m/z* calcd for C_19_H_34_N_5_O_2_, 364.27070 [M + H]^+^; found: 364.27069.

*N-(1-((E)-3,7-Dimethylocta-2,6-dien-1-yl)-5-phenyl-1,3,5-triazinan-2-ylidene)nitramide* (**4j**), white solid, m.p. 108–110 °C, yield 47.6%. IR ν/cm^−1^: 3284, 2969, 2911, 2847, 1714, 1668, 1580, 1551, 1493, 1432, 1382, 1314; ^1^H-NMR: δ ppm 9.75 (s, 1H, Het-NH), 7.30–7.36 (m, 2H, Ar-H), 7.02–7.12 (m, 3H, Ar-H), 5.05–5.08 (m, 2H, C=CH), 4.90–4.91 (m, 2H, Het-H), 4.76 (s, 2H, Het-H), 4.11 (d, 2H, *J* = 7.14 Hz, Het-CH_2_), 2.01–2.07 (m, 4H, C-CH_2_CH_2_-C), 1.67–1.68 (m, 6H, C-(CH_3_)_2_), 1.60 (s, 3H, C-CH_3_); ^13^C-NMR: δ ppm 155.32, 146.21, 141.63, 131.50, 129.24, 123.31, 123.12, 118.90, 117.27, 63.75, 59.30, 43.61, 39.15, 25.93, 25.31, 17.35, 15.97. HRMS (ESI^+^) *m/z* calcd for C_19_H_28_N_5_O_2_, 358.22375 [M + H]^+^; found: 358.22360.

*N-(1-((E)-3,7-Dimethylocta-2,6-dien-1-yl)-5-benzyl-1,3,5-triazinan-2-ylidene)nitramide* (**4k**), white solid, m.p. 76–77 °C, yield 55.6%. IR ν/cm^−1^: 3274, 3027, 2966, 2921, 2857, 1670, 1585, 1553, 1367, 1291; ^1^H-NMR: δ ppm 9.59 (brs, 1H, Het-NH), 7.31–7.37 (m, 5H, Ar-H), 5.03–5.16 (m, 2H, C=CH), 4.36–4.37 (m, 2H, Het-H), 4.22 (s, 2H, Het-H), 4.04 (d, 2H, *J* = 7.17 Hz, Het-CH_2_), 3.84 (s, 2H, N-CH_2_), 2.01–2.07 (m, 4H, C-CH_2_CH_2_-C), 1.67 (s, 3H, C-CH_3_), 1.58 (d, 6H, *J* = 7.32 Hz, C-(CH_3_)_2_); ^13^C-NMR: δ ppm 154.89, 141.18, 136.07, 131.53, 128.59, 128.34, 127.67, 123.30, 117.51, 64.18, 59.79, 54.82, 43.62, 39.21, 25.92, 25.34, 17.35, 15.87. HRMS (ESI^+^) *m/z* calcd for C_20_H_30_N_5_O_2_, 372.23940 [M + H]^+^; found: 372.23917.

*N-(1-((E)-3,7-Dimethylocta-2,6-dien-1-yl)-5-(4-chlorobenzyl)-1,3,5-triazinan-2-ylidene)nitramide* (**4l**), white solid, m.p. 65–67 °C, yield 55.4%. IR ν/cm^−1^: 3283, 2982, 2967, 2914, 2888, 2853, 1588, 1546, 1435, 1387, 1297, 1244; ^1^H-NMR: δ ppm 9.50 (brs, 1H, Het-NH), 7.19–7.30 (m, 4H, Ar-H), 5.10–5.14 (m, 1H, C=CH), 4.98–5.03 (m, 1H, C=CH), 4.38 (s, 2H, Het-H), 4.18 (s, 2H, Het-H), 3.99 (d, 2H, *J* = 7.14 Hz, Het-CH_2_), 1.94–2.05 (m, 4H, C-CH_2_CH_2_-C), 1.62 (d, 3H, *J* = 0.78 Hz, C-CH_3_), 1.55–1.57 (m, 6H, C-CH_3_); ^13^C-NMR: δ ppm 154.78, 141.20, 134.84, 133.27, 131.50, 129.88, 128.39, 123.25, 117.49, 64.10, 59.84, 54.04, 43.51, 39.19, 25.90, 25.32, 17.33, 15.85. HRMS (ESI^+^) *m/z* calcd for C_20_H_29_ClN_5_O_2_, 406.20043 [M + H]^+^; found: 406.20068.

*N-(1-((E)-3,7-Dimethylocta-2,6-dien-1-yl)-5-(2-methylphenyl)-1,3,5-triazinan-2-ylidene)nitramide* (**4m**), white solid, m.p. 72–74 °C, yield 29.2%. IR ν/cm^−1^: 3306, 3025, 2965, 2925, 1669, 1591, 1544, 1494, 1423, 1386, 1276; ^1^H-NMR: δ ppm 9.72 (brs, 1H, Het-NH), 7.06–7.23 (m, 4H, Ar-H), 5.02–5.04 (m, 2H, C=CH), 4.71 (s, 2H, Het-H), 4.58 (s, 2H, Het-H), 4.11 (d, 2H, *J* = 7.27 Hz, Het-CH_2_), 2.30 (s, 3H, Ar-CH_3_), 1.95–2.04 (m, 4H, C-CH_2_CH_2_-C), 1.64 (d, 6H, *J* = 4.21 Hz, C-(CH_3_)_2_), 1.67 (s, 3H, C-CH_3_); ^13^C-NMR: δ ppm 155.51, 145.73, 141.62, 132.14, 131.49, 131.19, 126.78, 125.24, 123.30, 120.73, 117.06, 64.33, 59.95, 43.48, 39.13, 25.91, 25.28, 17.50, 17.35, 15.92. HRMS (ESI^+^) *m/z* calcd for C_20_H_30_N_5_O_2_, 372.23940 [M + H]^+^; found: 372.23889.

*N-(1-((E)-3,7-Dimethylocta-2,6-dien-1-yl)-5-(3-methylphenyl)-1,3,5-triazinan-2-ylidene)nitramide* (**4n**), white solid, m.p. 101–102 °C, yield 25.8%. IR ν/cm^−1^: 3283, 3012, 2969, 2916, 2847, 1666, 1606, 1578, 1551, 1492, 1430, 1383, 1290; ^1^H-NMR: δ ppm 9.53 (brs, 1H, Het-NH), 7.18 (t, 1H, *J* = 7.82 Hz, Ar-H), 6.91–6.96 (m, 2H, Ar-H), 6.81 (d, 1H, *J* = 7.23 Hz, Ar-H), 5.00–5.05 (m, 2H, C=CH), 4.90 (d, 4H, *J* = 6.53 Hz, Het-H), 3.95 (d, 2H, *J* = 6.85 Hz, Het-CH_2_), 2.27 (s, 3H, Ar-CH_3_),1.96–2.02 (m, 4H, C-CH_2_CH_2_-C), 1.68 (s, 3H, CH_3_) , 1.62 (s, 3H, CH_3_) , 1.55 (s, 3H, CH_3_); ^13^C-NMR: δ ppm 155.38, 146.21, 141.65, 139.18, 131.58, 129.09, 124.05, 123.27, 119.63, 117.30, 115.95, 63.68, 59.43, 43.64, 39.21, 25.95, 25.32, 21.27, 17.36, 15.99. HRMS (ESI^+^) *m/z* calcd for C_20_H_30_N_5_O_2_, 372.23940 [M + H]^+^; found: 372.23911.

*N-(1-((E)-3,7-Dimethylocta-2,6-dien-1-yl)-5-(4-methylphenyl)-1,3,5-triazinan-2-ylidene)nitramide* (**4o**), white solid, m.p. 135–136 °C, yield 30.1%. IR ν/cm^−1^: 3278, 3010, 2968, 2914, 2853, 1580, 1551, 1497, 1432, 1382, 1286; ^1^H-NMR: δ ppm 9.71 (brs, 1H, Het-NH), 7.12 (d, 2H, *J* = 8.40 Hz, Ar-H), 6.94 (d, 2H, *J* = 8.49 Hz, Ar-H), 5.05–5.07 (m, 2H, C=CH), 4.86 (s, 2H, Het-H), 4.71 (s, 2H, Het-H), 4.09 (d, 2H, *J* = 7.17 Hz, Het-CH_2_), 2.30 (s, 3H, Ar-CH_3_), 2.02–2.06 (m, 4H, C-CH_2_CH_2_-C), 1.67 (s, 6H, C-(CH_3_)_2_), 1.60 (s, 3H, C-CH_3_); ^13^C-NMR: δ ppm 155.37, 143.87, 141.60, 133.02, 131.56, 129.82, 123.32, 119.19, 117.26, 64.12, 59.70, 43.69, 39.18, 25.94, 25.32, 20.23, 17.36, 16.01. HRMS (ESI^+^) *m/z* calcd for C_20_H_30_N_5_O_2_, 372.23940 [M + H]^+^; found: 372.23901.

*N-(1-((E)-3,7-Dimethylocta-2,6-dien-1-yl)-5-(4-ethylphenyl)-1,3,5-triazinan-2-ylidene)nitramide* (**4p**), white solid, m.p. 76–78 °C, yield 43.1%. IR ν/cm^−1^: 3286, 2967, 2929, 2857, 1579, 1546, 1532, 1380, 1240; ^1^H-NMR: δ ppm 9.72 (brs, 1H, Het-NH), 7.15–7.19 (m, 2H, Ar-H), 6.96–7.01 (m, 2H, Ar-H), 5.06–5.12 (m, 2H, C=CH), 4.90 (t, 2H, *J* = 1.08 Hz, Het-H), 4.75 (s, 2H, Het-H), 4.12 (d, 2H, *J* = 7.17 Hz, Het-CH_2_), 2.63 (q, 2H, *J* = 7.62 Hz, Ar-CH_2_), 2.03–2.10 (m, 4H, C-CH_2_CH_2_-C), 1.70 (s, 6H, C-CH_3_), 1.63 (s, 3H, C-CH_3_), 1.24 (t, 3H, *J* = 7.59 Hz, C-CH_3_); ^13^C-NMR: δ ppm 155.39, 144.03, 141.64, 139.50, 131.58, 128.65, 123.30, 119.28, 117.27, 64.10, 59.76, 43.68, 39.20, 27.71, 25.96, 25.32, 17.36, 15.99, 15.19. HRMS (ESI^+^) *m/z* calcd for C_21_H_32_N_5_O_2_, 386.25505 [M+H]^+^; found: 386.25449.

*N-(1-((E)-3,7-Dimethylocta-2,6-dien-1-yl)-5-(4-fluorophenyl)-1,3,5-triazinan-2-ylidene)nitramide* (**4q**), white solid, m.p. 101–103 °C, yield 59.2%. IR ν/cm^−1^: 3281, 2975, 2933, 2915, 2847, 1580, 1552, 1533, 1382, 1285, 1237; ^1^H-NMR: δ ppm 9.73 (brs, 1H, Het-NH), 7.03–7.05 (m, 4H, Ar-H), 5.05–5.10 (m, 2H, C=CH), 4.89 (t, 2H, *J* = 1.05 Hz, Het-H), 4.72 (s, 2H, Het-H), 4.11 (d, 2H, *J* = 7.20 Hz, Het-CH_2_), 1.98–2.08 (m, 4H, C-CH_2_CH_2_-C), 1.69 (s, 6H, C-CH_3_), 1.62 (s, 3H, C-CH_3_); ^13^C-NMR: δ ppm 158.89 (d, *J*_C-F_ = 242.36 Hz), 155.39, 142.67 (d, *J*_C-F_ = 2.81 Hz), 141.92, 131.68, 123.16, 121.22 (d, *J*_C-F_ = 8.03 Hz), 117.07, 115.96 (d, *J*_C-F_ = 22.50 Hz), 64.39, 60.00, 43.65, 39.18, 25.94, 25.30, 17.33, 15.99. HRMS (ESI^+^) *m/z* calcd for C_19_H_27_FN_5_O_2_, 376.21433 [M + H]^+^; found: 376.21405.

*N-(1-((E)-3,7-Dimethylocta-2,6-dien-1-yl)-5-(4-chlorophenyl)-1,3,5-triazinan-2-ylidene)nitramide* (**4r**), white solid, m.p. 134–136 °C, yield 20.3%. IR ν/cm^−1^: 3391, 3278, 2969, 2912, 2852, 1579, 1552, 1493, 1434, 1383, 1287, 821; ^1^H-NMR: δ ppm 9.56 (brs, 1H, Het-NH), 7.32–7.36 (m, 2H, Ar-H), 7.15–7.19 (m, 2H, Ar-H), 5.04–5.05 (m, 2H, C=CH), 4.91–4.97 (m, 4H, Het-H), 3.94 (d, 2H, *J* = 6.87 Hz, Het-CH_2_), 1.91–2.00 (m, 4H, C-CH_2_CH_2_-C), 1.66 (s, 3H, C-CH_3_) , 1.62 (s, 3H, C-CH_3_) , 1.55 (s, 3H, C-CH_3_); ^13^C-NMR: δ ppm 155.34, 144.88, 142.04, 131.69, 129.28, 128.54, 123.18, 120.42, 117.06, 63.79, 59.44, 43.68, 39.19, 25.95, 25.32, 17.36, 16.01. HRMS (ESI^+^) *m/z* calcd for C_19_H_27_ClN_5_O_2_, 392.18478 [M + H]^+^; found: 392.18484.

*N-(1-((E)-3,7-Dimethylocta-2,6-dien-1-yl)-5-(4-methoxyphenyl)-1,3,5-triazinan-2-ylidene)nitramide* (**4s**), white solid, m.p. 91–93 °C, yield 38.5%. IR ν/cm^−1^: 3291, 3062, 2965, 2908, 2854, 1672, 1594, 1542, 1511, 1457, 1385, 1292; ^1^H-NMR: δ ppm 9.52 (brs, 1H, Het-NH), 7.04–7.08 (m, 2H, Ar-H), 6.85–6.90 (m, 2H, Ar-H), 5.02–5.06 (m, 1H, C=CH), 4.93–4.97 (m, 1H, C=CH), 4.83 (d, 4H, *J* = 8.78 Hz, Het-H), 3.94 (d, 2H, *J* = 6.83 Hz, Het-CH_2_), 3.70 (s, 3H, Ar-OCH_3_), 1.91–2.00 (m, 4H, C-CH_2_CH_2_-C), 1.66 (s, 3H, C-CH_3_) , 1.62 (s, 3H, C-CH_3_) , 1.55 (s, 3H, C-CH_3_); ^13^C-NMR: δ ppm 155.81, 155.32, 141.52, 139.87, 131.50, 123.30, 121.03, 117.26, 114.42, 64.64, 60.11, 55.12, 43.60, 39.15, 25.94, 25.30, 17.33, 15.96. HRMS (ESI^+^) *m/z* calcd for C_20_H_30_N_5_O_3_, 388.23432 [M + H]^+^; found: 388.23370.

*N-(1-((E)-3,7-Dimethylocta-2,6-dien-1-yl)-5-(2,4-dimethylphenyl)-1,3,5-triazinan-2-ylidene)nitramide* (**4t**), white solid, m.p. 88–90 °C, yield 16.3%. IR ν/cm^−1^: 3292, 2960, 2928, 2856, 1666, 1592, 1552, 1507, 1458, 1380, 1269; ^1^H-NMR: δ ppm 9.48 (brs, 1H, Het-NH), 7.03 (s, 1H, Ar-H), 6.94 (s, 2H, Ar-H), 4.94–5.04 (m, 2H, C=CH), 4.60–4.66 (s, 4H, Het-H), 3.96 (d, 2H, *J* = 6.99 Hz, Het-CH_2_), 2.21 (s, 6H, Ar-CH_3_), 1.87–1.99 (m, 4H, C-CH_2_CH_2_-C), 1.62 (s, 3H, C-CH_3_) , 1.60 (s, 3H, C-CH_3_) , 1.55 (s, 3H, C-CH_3_); ^13^C-NMR: δ ppm 155.53, 143.26, 141.49, 134.90, 131.99, 131.84, 131.47, 127.23, 123.34, 120.71, 117.12, 64.57, 60.10, 43.52, 39.14, 25.92, 25.26, 20.32, 17.38, 17.32, 15.93. HRMS (ESI^+^) *m/z* calcd for C_21_H_32_N_5_O_2_, 386.25505 [M + H]^+^; found: 386.25500.

### 3.3. X-ray Diffraction

Single crystals of **4r** were C_19_H_26_ClN_5_O_2_ (CCDC 1437627 contains the supplementary crystallographic data for this paper. These data can be obtained free of charge via http://www.ccdc.cam.ac.uk/conts/retrieving.html (or from the CCDC, 12 Union Road, Cambridge CB2 1EZ, UK; Fax: +44 1223 336033; E-mail: deposit@ccdc.cam.ac.uk), shown in Figure 1). A suitable crystal was selected an analyzed on a SuperNova, Dual, Cu at zero, Atlas diffractometer (Agilent, CA, USA) PW. The crystal was kept at 180.01(10) K during data collection. Using Olex2 [30], the structure was solved with the Superflip [31] structure solution program using Charge Flipping and refined with the Shelx [32] refinement package using Least Squares minimisation.

### 3.4. Stability Test

The EβF and analogues **4r** were dissolved in methanol (chromatographically pure), respectively. After exposure to air for 48 h at room temperature, their changes of content were analyzed by high performance liquid chromatography (HPLC) on a LC-1AT HPLC instrument (Shimadzu). Chromatographic experiments were performed on C18 reversed-phase column (4.5 mm × 250 mm, 5 μm), the mobile phase was methanol and water (80:20); the detection wavelength was 245 nm, column temperature was 25 °C, the flow rate was 0.7 mL/min and the injection volume was 5 μL.

### 3.5. Biological Activity Test

#### 3.5.1. Repellent Assays

The repellent response of *Myzus persicae* to the EβF analogues was investigated with a glass T-tube (a two-way olfactometer) olfactometer [33] (one arm was used as “treatment” arm while the other was as “control” arm). A total air flow 0.4 L/min (each arm was 0.2 L/min) was introduced into the olfactometer, which firstly went through activated carbon to purify air flow and then pass through distilled water. Thus two well separated and purified air flow went continuously through the olfactometer arms. As a “treatment” arm, standard solutions (2.5 μL, dissolved in hexane with final concentration 2 × 10^−5^ g/10 μL) of the test compounds were applied to filter paper strips (1 cm^2^ diameter). The solvent was allowed to evaporate for 30 s before the filter paper strip was placed in the glass stimulus chamber. For control, 2.5 μL *n*-hexane as solvent was placed in the chamber of “control” arm and operated same as “treatment” arm. The whole olfactometer was washed with ethanol and hexane and dried by air before each test.

Apterous adult *Myzus persicae* were used in this assay. A total of twenty aphids were introduced at the center of the olfactometer arms and allowed freedom to walk toward either arm. After 15 min, the number of aphids, which moved more than 2 cm distance to the olfactometer center, was recorded as treatment arm or control arm. The experiment was replicated 10 times with each analogue. The repellent activity of each EBF analogue was estimated by the repellent proportion (RP), the modification of excess proportion index [34], calculated by the formula RP = C/(C + T) × 100%, where T represents the number of aphids in the arm treated with the tested solutions and C indicates those in the control arm. The numbers of aphids in control and treatment arm were compared statistically with that of SPSS Statistics version 21 (IBM) by paired t-test. Also the repellent proportion of the EβF analogues was analyzed statistically with that of SPSS Statistics version 21 (IBM) using one-way analysis of variance (ANOVA) followed by Duncan’s test at *p* < 0.05.

#### 3.5.2. Aphicidal Assays

The aphicidal activity of the EβF analogues against *Myzus* *persicae* was evaluated using the reported procedure [28]. All the test compounds were dissolved in acetone to a concentration of 3000 μg/mL and then diluted with 0.5% Tween 80 to the test concentration 300 μg/mL. Soybean plant leaf discs of about 3 cm diameter were dipped into the test solutions for 10 s. Using the same test concentration, the discs dipped into EβF solution were set as the positive control while the discs dipped into 0.5% Tween 80 were set as the negative control. After air-drying, the treated leaf discs were placed individually into bioassay polyvinyl plates (10 cm × 13 cm × 2.5 cm, each plate had twelves (3 × 4) holes of 3.0 cm diameter each) with 1% agar to keep moist. And then, the discs were infested with 20 ± 3 apterous adult aphids and kept in an incubator with constant temperature (25 ± 1 °C) and light period (light:dark = 8:16) for 48 h. Each treatment was performed six times. The number of dead aphids was counted, and then mortality rates were corrected using Abbott’s formula [35]. The aphicidal activity of the EβF analogues were compared with that of SPSS Statistics version 21 (IBM) using one way analysis of variance (ANOVA) followed by Duncan’s test at *p* < 0.05.

## 4. Conclusions

In summary, a series of novel (*E*)-β-farnesene analogues were designed by replacing the conjugated double bonds of EβF with 2-nitroiminohexahydro-1,3,5-triazine. All the title compounds were synthesized via the nucleophilic substitution reaction of intermediates **2** and **3**. The performed bioassay proved that some analogues, such as **4r** and **4t**, showed good repellent activity against *Myzus persicae*. Particularly, the analogue **4r**, with more stability than the lead EβF, also exhibited similar aphicidal activity as pymetrozine. Therefore, **4r** would be a promising lead for the further optimization. In the meantime, the structure-activity relationship provided the valuable clues for the design of new EβF analogues. Introducing phenyl rather than alkyl, cyclohexyl or benzyl at 5 positon of 2-nitroiminohexahydro-1,3,5-triazine is strongly recommended and it is favorable to use electron-withdrawing groups in the *para*-position or disubstitution in the benzene ring.

## Figures and Tables

**Table 1:** Crystal data and structure refinement for **4r**.

Compound	4r
Empirical formula	C_10_H_13_N_5_O_3_
Formula weight	251.25
Temperature/K	180.15
Crystal system	monoclinic
Space group	P2_1_/c
a/Å	15.4268(13)
b/Å	5.3261(4)
c/Å	14.6589(11)
α/°	90
β/°	110.045(9)
γ/°	90
Volume/Å^3^	1131.48(16)
Z	4
ρ _calc_ mg/mm^3^	1.475
m/mm^−1^	0.113
F(000)	528.0
Crystal size/mm^3^	0.2 × 0.1 × 0.1
2Θ range for data collection	7.372° to 50.036°
Index ranges	−15 ≤ h ≤ 16, −6 ≤ k ≤ 3, −14 ≤ l ≤ 17
Reflections collected	2423
Independent reflections	1780[R(int) = 0.0173]
Data/restraints/parameters	1780/0/164
Goodness-of-fit on F^2^	1.086
Final R indexes (I ≥ 2σ (I))	R_1_ = 0.0371, wR_2_ = 0.0914
Final R indexes [all data]	R_1_ = 0.0480, wR_2_ = 0.0996
Largest diff. peak/hole/e Å^−3^	0.16/−0.22
CCDC No.	1437627

**Table 2:** Repellent proportion (RP) of the EβF analogues **4a**–**4t** against *M. persicae*.

Compd.	R	RP (%) ^a^	Compd.	R	RP (%) ^a^
**4a**	CH_3_	52.28 ± 2.40 ^a^	**4l**	(4-Cl)-benzyl	67.56 ± 2.77 ^f,g^
**4b**	CH_2_CH_3_	56.74 ± 1.37 ^b^	**4m**	(2-CH_3_)-phenyl	75.62 ± 2.16 ^h^
**4c**	(CH_2_)_2_CH_3_	60.89 ± 1.41 ^c^	**4n**	(3-CH_3_)-phenyl	67.28 ± 1.66 ^f^
**4d**	(CH_2_)_3_CH_3_	64.03 ± 1.40 ^e^	**4o**	(4-CH_3_)-phenyl	69.47 ± 1.91 ^g^
**4e**	(CH_2_)_4_CH_3_	55.96 ± 1.54 ^b^	**4p**	(4-CH_2_CH_3_)-phenyl	67.79 ± 1.81 ^f,g^
**4f**	CH(CH_3_)_2_	61.34 ± 1.42 ^c^	**4q**	(4-F)-phenyl	74.11 ± 2.74 ^h^
**4g**	CHCH_2_(CH_3_)_2_	63.89 ± 1.42 ^d,e^	**4r**	(4-Cl)-phenyl	78.43 ± 2.00 ^i^
**4h**	C(CH_3_)_3_	62.89 ± 1.48 ^c–−e^	**4s**	(4-OCH_3_)-phenyl	67.89 ± 2.24 ^f,g^
**4i**	cyclohexyl	67.16 ± 2.09 ^f^	**4t**	(2,4-(CH_3_)_2_)-phenyl	80.07 ± 2.20 ^i^
**4j**	phenyl	62.57 ± 1.71 ^c–−e^	**EβF**	-	96.47 ± 2.31 ^j^
**4k**	benzyl	61.93 ± 1.69 ^c,d^			

a: The repellent activity of each EBF analogue was estimated by the repellent proportion (RP), calculated by the formula RP = C/(C + T) × 100%, where T represents the number of aphids in the treatment arm and C indicates those in the control arm. Values are mean ± standard deviation. To determine significant differences in the mortality of the EβF analogues, analysis of variance (One-way ANOVA) was performed followed by Duncan’s test (*p* < 0.05). Different letters indicate significant differences. *F*-value = 28.05; df1 (degrees of freedom between groups) = 20; df2 (degrees of freedom within groups) = 189; *p* < 0.001.

**Table 3:** Aphicidal activity of the EβF analogues **4a**–**4t** at 300 μg/mL.

Compd.	R	Mortality (%) ^a^ (48 h)	Compd.	R	Mortality (%) ^a^ (48 h)
**4a**	CH_3_	73.32 ± 3.28 ^c^	**4l**	(4-Cl)-benzyl	72.46 ± 2.79 ^c^
**4b**	CH_2_CH_3_	78.26 ± 3.53 ^d,e^	**4m**	(2-CH_3_)-phenyl	42.84 ± 3.09 ^a^
**4c**	(CH_2_)_2_CH_3_	86.49 ± 2.46 ^f,g^	**4n**	(3-CH_3_)-phenyl	44.91 ± 1.74 ^a^
**4d**	(CH_2_)_3_CH_3_	83.22 ± 3.74 ^e,f^	**4o**	(4-CH_3_)-phenyl	79.14 ± 2.69 ^e^
**4e**	(CH_2_)_4_CH_3_	80.18 ± 4.66 ^e^	**4p**	(4-CH_2_CH_3_)-phenyl	80.89 ± 2.57 ^e^
**4f**	CH(CH_3_)_2_	73.85 ± 3.51 ^c,d^	**4q**	(4-F)-phenyl	88.86 ± 4.71 ^g^
**4g**	CHCH_2_(CH_3_)_2_	70.63 ± 4.28 ^c^	**4r**	(4-Cl)-phenyl	82.05 ± 3.74 ^e,f^
**4h**	C(CH_3_)_3_	63.26 ± 2.80 ^b^	**4s**	(4-OCH_3_)-phenyl	80.83 ± 2.68 ^e^
**4i**	cyclohexyl	61.57 ± 3.24 ^b^	**4t**	(2,4-(CH_3_)_2_)-phenyl	74.05 ± 5.33 ^c,d^
**4j**	phenyl	60.78 ± 3.17 ^b^	**EβF**	-	60.25 ± 4.42 ^b^
**4k**	benzyl	59.69 ± 3.43 ^b^	**Pymetrozine**	-	80.68 ± 3.83 ^e^

^a^: Values are mean ± standard deviation mortality of EβF analogues against aphid; to determine significant differences in the mortality of the EβF analogues, analysis of variance (One-way ANOVA) was performed followed by Duncan’s test (*p* < 0.05). Different letters indicate significant differences. *F*-value = 62.74; df1 (degrees of freedom between groups) = 21; df2 (degrees of freedom within groups) = 110; *p* < 0.001.

## Supplementary Materials

Supplementary materials can be accessed at: http://www.mdpi.com/1420-3049/21/7/825/s1.
