# Supplementary material for: Novel (E)-β-Farnesene Analogues Containing 2-Nitroiminohexahydro-1,3,5-triazine: Synthesis and Biological Activity Evaluation
Source: Molecules. 2016 Jun 24;21(7):825. doi: 10.3390/molecules21070825 (PMC6273983; doi:10.3390/molecules21070825)
Supplement: Supplementary file 1 [file molecules-21-00825-s001.pdf]

Supplementary Materials: Novel (*E*)-β-Farnesene Analogues Containing 2-Nitroiminohexahydro-1,3,5-triazine: Synthesis and Biological Activities Evaluation

Yaoguo Qin, Jingpeng Zhang, Dunlun Song, Hongxia Duan, Wenhao Li and Xinling Yang

Crystal Structure of 4r

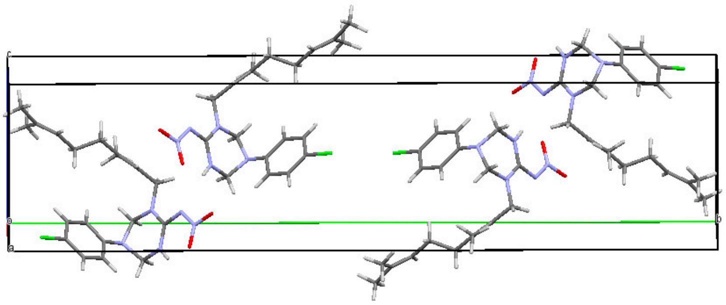

Figure S1. Crystal Packing of Compound **4r**.

Table S1. Fractional Atomic Coordinates (×10^4) and Equivalent Isotropic Displacement Parameters (Å^2 × 10^3) for **4r**.

Atom	*x*	*y*	*z*	U(eq)	Atom	*x*	*y*	*z*	U(eq)
C19	8210(20)	5271(3)	−1128(16)	267(10)	C14	6270(12)	6309.7(14)	−617(6)	61.8(17)
C1	4206(9)	7206.2(11)	4113(5)	31.3(11)	C15	6266(16)	5945.6(17)	−56(7)	98(3)
C2	5216(9)	6908.3(12)	6426(5)	39.2(13)	C16	5150(20)	5694(2)	−1176(9)	127(4)
C3	7230(9)	6767.3(13)	4389(5)	43.4(13)	C17	5930(16)	5405.1(19)	−1647(8)	87(2)
C4	4269(9)	6356.4(12)	5133(5)	36.7(12)	C18	4754(16)	5183.3(19)	−2816(8)	111(3)
C5	2222(9)	6332.2(13)	5794(5)	41.5(13)	Cl1	−628(3)	5462.4(4)	4066(2)	87.4(7)
C6	726(10)	6058.0(13)	5488(6)	48.6(14)	N1	5876(7)	7030.9(10)	3535(4)	36.9(10)
C7	1230(10)	5808.5(13)	4481(7)	54.3(15)	N2	3761(7)	7143.8(9)	5493(4)	34.2(10)
C8	3241(11)	5832.8(15)	3779(7)	61.9(17)	N3	5948(7)	6618.8(10)	5530(4)	36.6(10)
C9	4759(10)	6104.0(13)	4115(6)	48.3(14)	N4	3068(8)	7439.9(10)	3188(4)	36.7(10)
C10	6667(9)	7099.4(12)	2055(5)	39.5(13)	N5	1275(7)	7620.2(10)	3676(4)	36.6(10)
C11	5971(10)	6822.7(13)	1006(5)	45.8(14)	O1	328(7)	7550.6(9)	4811(4)	51.4(10)
C12	7292(11)	6576.3(13)	486(5)	48.0(15)	O2	543(6)	7864.3(9)	2855(3)	47.5(10)
C13	9839(11)	6542.7(16)	869(7)	67.9(19)					

Table S2. Anisotropic Displacement Parameters (Å^2 × 10^3) for **4r**.

Atom	U_{11}	U_{22}	U_{33}	U_{23}	U_{13}	U_{12}
C19	209(15)	193(12)	370(20)	−138(13)	−184(15)	96(12)
C1	32(3)	32(3)	30(2)	2(2)	5(2)	7(2)
C2	43(3)	42(3)	30(2)	−1(2)	−8(2)	1(3)
C3	30(3)	46(3)	54(3)	3(3)	6(3)	1(3)
C4	33(3)	37(3)	40(3)	2(2)	−2(2)	12(2)
C5	38(3)	41(3)	45(3)	−2(2)	−1(3)	10(3)
C6	39(4)	44(3)	64(3)	2(3)	9(3)	0(3)
C7	38(4)	34(3)	90(4)	−5(3)	−2(3)	3(3)
C8	55(4)	49(4)	82(4)	−21(3)	7(4)	0(3)
C9	39(4)	47(3)	60(3)	−13(3)	9(3)	1(3)
C10	38(3)	43(3)	39(3)	1(2)	11(2)	4(3)
C11	42(4)	49(3)	46(3)	−1(3)	1(3)	4(3)
C12	58(4)	41(3)	47(3)	0(2)	13(3)	−1(3)
C13	49(4)	66(4)	92(5)	−21(3)	23(4)	19(3)

Table S2. *Cont.*

Atom	U_{11}	U_{22}	U_{33}	U_{23}	U_{13}	U_{12}
C14	75(5)	59(4)	51(3)	−6(3)	6(3)	3(4)
C15	157(9)	56(4)	78(4)	−4(4)	−18(5)	−13(5)
C16	180(10)	67(5)	122(7)	−18(5)	−65(7)	14(6)
C17	104(7)	66(5)	86(5)	−12(4)	−28(5)	2(5)
C18	139(8)	76(5)	112(6)	−30(5)	−33(6)	−7(5)
Cl1	60.4(12)	49.4(10)	152.2(17)	−23.2(11)	5.2(12)	−11.3(9)
N1	40(3)	32(2)	40(2)	3.9(18)	6(2)	7(2)
N2	36(3)	38(2)	28.9(19)	−1.2(17)	4.6(19)	2(2)
N3	36(3)	38(2)	36(2)	3.8(18)	0(2)	−3(2)
N4	40(3)	40(2)	31(2)	−1.2(18)	6(2)	5(2)
N5	33(3)	46(3)	30(2)	−2(2)	−2(2)	2(2)
O1	55(3)	63(2)	39.3(19)	8.0(17)	19(2)	10(2)
O2	49(2)	53(2)	41.1(18)	11.7(17)	2.8(18)	14.7(19)

The anisotropic displacement factor exponent takes the form: $-2\pi^2 \times (h^2a^2 \times U_{11} + ... + 2hka \times b \times U_{12})$

Table S3. Bond Lengths for **4r**.

Atom	Atom	Length/Å	Atom	Atom	Length/Å	Atom	Atom	Length/Å	Atom	Atom	Length/Å
C19	C17	1.470(13)	C3	N3	1.442(6)	C7	Cl1	1.734(6)	C14	C15	1.482(8)
C1	N1	1.323(6)	C4	C5	1.379(7)	C8	C9	1.383(7)	C15	C16	1.516(9)
C1	N2	1.326(5)	C4	C9	1.384(6)	C10	C11	1.464(6)	C16	C17	1.280(10)
C1	N4	1.367(5)	C4	N3	1.430(6)	C10	N1	1.483(5)	C17	C18	1.490(9)
C2	N2	1.467(6)	C5	C6	1.379(7)	C11	C12	1.325(7)	N4	N5	1.354(5)
C2	N3	1.458(6)	C6	C7	1.371(7)	C12	C13	1.506(8)	N5	O1	1.239(5)
C3	N1	1.468(6)	C7	C8	1.380(8)	C12	C14	1.523(7)	N5	O2	1.252(5)

Table S4. Bond Angles for **4r**.

Atom	Atom	Atom	Angle/°	Atom	Atom	Atom	Angle/°	Atom	Atom	Atom	Angle/°
N1	C1	N2	119.2(4)	C7	C8	C9	120.0(5)	C1	N1	C3	121.2(4)
N1	C1	N4	115.0(4)	C8	C9	C4	120.7(5)	C1	N1	C10	124.1(4)
N2	C1	N4	125.7(4)	C11	C10	N1	112.3(4)	C3	N1	C10	114.4(4)
N3	C2	N2	108.7(3)	C12	C11	C10	127.3(5)	C1	N2	C2	121.1(4)
N3	C3	N1	111.5(4)	C11	C12	C13	124.5(5)	C3	N3	C2	107.2(4)
C5	C4	C9	118.4(5)	C11	C12	C14	120.1(6)	C4	N3	C2	116.8(4)
C5	C4	N3	122.3(4)	C13	C12	C14	115.3(5)	C4	N3	C3	118.3(4)
C9	C4	N3	119.2(5)	C15	C14	C12	114.1(5)	N5	N4	C1	118.8(4)
C6	C5	C4	121.1(5)	C14	C15	C16	111.9(5)	O1	N5	N4	124.6(4)
C7	C6	C5	120.1(5)	C17	C16	C15	128.9(9)	O1	N5	O2	120.5(4)
C6	C7	C8	119.7(5)	C19	C17	C18	113.0(8)	O2	N5	N4	114.8(4)
C6	C7	Cl1	121.0(5)	C16	C17	C19	121.9(8)				
C8	C7	Cl1	119.3(5)	C16	C17	C18	125.1(8)				

Table S5. Hydrogen Atom Coordinates (Å × 10^4) and Isotropic Displacement Parameters (Å^2 × 10^3) for **4r**.

Atom	*x*	*y*	*z*	U(eq)	Atom	*x*	*y*	*z*	U(eq)
H19A	9222	5274	−1937	401	H11	4396	6819	658	55
H19B	8056	5030	−777	401	H13A	10399	6752	1404	102
H19C	8859	5418	−324	401	H13B	10636	6519	−35	102
H2A	6576	7037	6859	47	H13C	10138	6335	1485	102
H2B	4344	6818	7236	47	H14A	4669	6379	−918	74
H3A	7682	6579	3725	52	H14B	7153	6317	−1503	74
H3B	8651	6877	4837	52	H15A	5428	5938	848	118
H5	1836	6508	6472	50	H15B	7869	5871	203	118
H6	−655	6042	5975	58	H16	3665	5759	−1583	152
H8	3583	5663	3066	74	H18A	3317	5296	−3185	167
H9	6154	6117	3641	58	H18B	4414	4953	−2409	167
H10A	6026	7325	1687	47	H18C	5757	5155	−3624	167
H10B	8364	7119	2128	47	H2	2571	7245	5854	41
